# A robust data-driven genomic signature for idiopathic pulmonary fibrosis with applications for translational model selection

**DOI:** 10.1371/journal.pone.0215565

**Published:** 2019-04-18

**Authors:** Ron Ammar, Pitchumani Sivakumar, Gabor Jarai, John Ryan Thompson

**Affiliations:** 1 Translational Bioinformatics, Translational Medicine, Bristol-Myers Squibb, Princeton, NJ, United States of America; 2 Fibrosis, Translational Research & Development, Bristol-Myers Squibb, Princeton, NJ, United States of America; Medizinische Hochschule Hannover, GERMANY

## Abstract

Idiopathic pulmonary fibrosis (IPF) is a chronic and progressive lung disease affecting ~5 million people globally. We have constructed an accurate model of IPF disease status using elastic net regularized regression on clinical gene expression data. Leveraging whole transcriptome microarray data from 230 IPF and 89 control samples from *Yang et al*. (2013), sourced from the Lung Tissue Research Consortium (LTRC) and National Jewish Health (NJH) cohorts, we identify an IPF gene expression signature. We performed optimal feature selection to reduce the number of transcripts required by our model to a parsimonious set of 15. This signature enables our model to accurately separate IPF patients from controls. Our model outperforms existing published models when tested with multiple independent clinical cohorts. Our study underscores the utility of elastic nets for gene signature/panel selection which can be used for the construction of a multianalyte biomarker of disease. We also filter the gene sets used for model input to construct a model reliant on secreted proteins. Using this approach, we identify the preclinical bleomycin rat model that is most congruent with human disease at day 21 post-bleomycin administration, contrasting with earlier timepoints suggested by other studies.

## Introduction

Idiopathic Pulmonary Fibrosis (IPF) is a fatal disease of unknown etiology characterized by scarring of the lung parenchyma resulting in progressive loss of lung function and eventual death [1]. Although two recently approved drugs, pirfenidone and nintedanib, reduce lung function decline in IPF, their efficacy is limited and mechanism of action poorly understood [2–4]. Even though meta analyses of large clinical trials suggest that pirfenidone reduces risk of mortality [5], lung transplant still remains the only option to significantly prolong survival in IPF, suggesting a dire need for new therapies. Development of new drugs for IPF is extremely challenging due to complicated diagnosis, limited disease understanding, lack of robust pre-clinical models predictive of human disease as well as biomarkers of disease progression and drug treatment. Current diagnosis of IPF requires careful integration of radiographic findings (honeycombing and presence of fibroblast foci), lung function (FVC, FEV1 and 6-minute walk test) and clinical data and the rational exclusion of other potentially similar interstitial lung diseases [6]. Often, the disease is diagnosed at an advanced stage when it is refractory to treatment. Therefore, there is a pressing need to develop newer, less-invasive and robust methods to efficiently diagnose IPF and enable early intervention strategies. Transcriptomic and proteomic disease signatures generated from clinically-relevant human samples including tissue and plasma, combined with robust in silico modeling can enable translational disease understanding, diagnosis and stratification of patients for effective drug treatments. Several studies have utilized microarray profiling of IPF-patient derived lung tissue to define genes and/or pathways that are differentially-regulated in comparison to healthy controls or patients with other lung diseases [4,7–9] and define signatures for disease classification. Peripheral blood profiling across small cohorts of patients have also identified potential biomarkers of disease such as MMP1 and MMP7 [10–12].

Comparative gene expression profiles of preclinical models of fibrosis with human tissue derived profiles have provided useful information on the utility of the models as well as insights into pathways or mechanisms that are altered during the induction, progression and resolution of fibrosis [13,14]. In many of these studies, gene/protein expression profiles have been correlated to clinical diagnosis, disease severity and measures of lung function [15].

In the most commonly studied preclinical model of IPF, the chemotherapeutic antibiotic bleomycin is intratracheally injected into rodents to induce an inflammatory response in the lung, damaging the epithelium, activating fibroblasts and ultimately leading to a fibrotic phase of increased collagen deposition and loss of alveolar structures [16]. The induced fibrosis manifests over the course of 7–14 days post-bleomycin treatment, and several studies have suggested different time points where congruence between the model and IPF are highest [16]. *Chaudhary et al*. (2006) measured profibrotic gene expression including pro-collagen I, TGF-*β*1, fibronectin and collagen deposition, determining the fibrotic phase to begin between days 9 and 14 post bleomycin treatment. *Bauer et al*. (2015) found the most congruent rat bleomycin model (day post-treatment) by first extracting a differential-expression signature from the rat and subsequently using that gene set to construct a translational signature from IPF samples. Day 7 was identified as having the highest similarity to IPF based on gene expression measurements [13]. The authors suggest that day 7 is the time point to administer antifibrotic compounds in order to best assess potential clinical outcomes.

Here, we have leveraged microarray profiling data from an extensive cohort of IPF and control samples within the Lung Tissue Research Consortium (LTRC) to develop an unbiased statistical model that defines a parsimonious 15-gene disease signature for IPF. The model has been trained and validated to accurately predict disease status across several IPF data sets. In addition, we identified a 29-gene secreted protein plasma signature for IPF and show that the bleomycin model of lung fibrosis at 21 days shows the largest congruence to the disease signature. Our work defines a robust genetic signature for IPF providing a potential multi-analyte biomarker panel for validation, as well as enables the identification of preclinical models that most closely resemble human IPF.

## Materials and methods

### Clinical data

Expression data for IPF and normal healthy patient lung samples were derived from 6 distinct cohorts (Table 1). The bulk of these expression data was available via the NCBI Gene Expression Omnibus (GEO). The clinical expression data include the Lung Tissue Research Consortium (LTRC; GSE32537) cohort [9,17], the Lung Genomics Research Consortium (LGRC; GSE47460) [17,18], the National Jewish Health (NJH) cohort [9] (data via personal communication, Ivana Yang) and several smaller cohorts, GSE10667 [7], GSE24206 [19] and GSE53845 [20]. Transcript abundances were measured on both Affymetrix and Agilent microarray platforms. The LGRC and LTRC share samples, and these were excluded appropriately during model testing. We also excluded non-IPF or normal patient samples such as non-IPF interstitial lung diseases and Chronic Obstructive Pulmonary Disease (COPD) (these can be found in the LGRC). We note that due to insufficient annotation information across all studies, we did not correct for cellular composition or type in lung tissue samples.

**Table 1:** Clinical samples.

Study	GEO Accession	Microarray platform	# IPF	# Normal
LTRC (Yang et al. (2013))	GSE32537	Affymetrix 1.0 ST	119	50
NJH (Yang et al. (2013))	NA	Affymetrix 1.0 ST	111	39
LGRC	GSE47460	Agilent-014850; Agilent-028004	160	108
Konishi et al. (2009)	GSE10667	Agilent-014850	23	15
Melzter et al. (2011)	GSE24206	Affymetrix U133	11	6
DePianto et al. (2015)	GSE53845	Agilent-014850	40	8

Models were trained and tested using these public cohorts of expression data for IPF and normal healthy patient lung samples. Sample counts are from the original studies.

In order to predict disease status of patients in the test cohorts we had to map Agilent expression measurements to the Affymetrix measurement space. This is similar to the scaling approach used by *Meltzer et al*. (2011) when mapping GSE24206 Affymetrix training features to the GSE10667 Agilent features. Conveniently, due to the common source of LTRC lung tissue used to generate both GSE32537 and GSE47460, there exist 85 common patient samples with both Affymetrix and Agilent data, allowing us to directly map expression signal across platforms. We generated gene-level scaling factors, which were possible because the ratios of Affymetrix/Agilent for each gene had very low variance. Genes included in the model were present on both Affymetrix and Agilent platforms.

### Preclinical model data

Bleomycin preclinical rat model data was publicly available (GSE48455) [13]. *Bauer et al*. (2013) intratracheally administered Sprague Dawley rats with a single instillation of saline or bleomycin and sacrified the animals along a time course of 3, 7, 14, 21, 28, 42, and 56 days post-treatment (Table 2). Rat-Human orthologs were mapped using NCBI HomoloGene [21]. For simplicity in interpretation, only orthologs with a one-to-one mapping were included (excluding one-to-many mappings).

**Table 2:** Bleomycin preclinical rat model samples.

	3	7	14	21	28	42	56
Bleomycin	5	5	5	5	5	5	5
Vehicle	5	5	4	5	5	5	5

Sample breakdown of bleomycin preclinical rat model (GSE48455) [13]. The time course experiment contains samples from 3, 7, 14, 21, 28, 42, and 56 days post-treatment.

### Identifying secreted proteins

Secreted genes were annotated using Gene Ontology (GO) cellular component annotations [22,23]. Genes were included if identified as existing in the extracellular space (GO:0005615) and not on the cell surface (GO:0009986). Our motivation was to exclude genes found on the cellular surface which were annotated as secreted.

### Computational and statistical processing

R version 3.4.1 and Bioconductor were used for expression data retrieval from GEO, normalization, filtering and scaling [24,25]. When present, batch/microarray platform effects were removed using the sva package [26]. Differential expression contrasts were computed using the limma package [27]. Regularized regression using elastic nets was computed using the glmnet package [28]. Balanced and repeated cross-validation was executed using the caret package [29].

All code and data required to execute the analysis described in this manuscript have been deposited in GitHub (https://github.com/ronammar/ipf_signature_elastic_net).

### Model construction and optimization

Disease status (IPF or normal) was used as a categorial response with two possible outcomes in logistic regression, which models the probability of response using a binomial link function to define a model of disease. However, logistic regression can be unreliable when *n*≈*p* or *p*>*n*. By linearly combining both *l*_1_ and *l*_2_ penalties of the lasso and ridge regression methods, respectively, *elastic net* regularization improves model performance and simultaneously selects features [28,30–32] (Appendix A1). Regularized regression techniques shrink coefficient estimates towards zero, and the use of the *l*_2_ penalty in our model forces some coefficient estimates to be equal to exactly zero. Coefficient estimates that are non-zero are selected for inclusion in the model [32].

All models were trained on the LTRC lung tissue expression data. Only gene expression data were used for modeling, and clinical or demographic data were not included as these covariates are not always available and of uniform quality. Disease classification was accomplished using an elastic net regularized regression model [28]. Elastic net training requires the selection of both a lasso and ridge mixing parameter, *α*, and a penalty strength parameter, *λ* (Appendix A1). To identify the optimal combination with the highest performance, we conducted 10-fold balanced cross-validation for each *α*,*λ* pair in a grid search on the LTRC training data (S1 Fig).

The grid search appears to indicate no significant performance associated with *α*, which controls the number of features included in the model. This means we can increase *α* to make the model more lasso-like, while maintaining high performance by adjusting *λ* accordingly. We chose *α* = 0.95 based on the suggestion in the glmnet documentation to set *α* = 1−*ϵ* for some small *ϵ*>0 [31]. The rationale is to improve numerical stability and reduce the degeneracies cause by high correlations between covariates.

Once we set *α*, we performed 1000 repeats of 10-fold cross-validation in caret to select the *λ* that yielded the highest performing model (lowest misclassification error) on the LTRC training data. This generated the final model and set of selected features (genes). For completeness, we computed the inclusion frequencies for each feature (S1 Supporting Information) [33,34]. We do not calculate significance of features in our model, as this is a relatively new and active area of statistics research [35]. Due to the challenges in computing appropriate estimates of the degrees of freedom, this significance test is currently in development for elastic nets.

This same approach was used to construct a model for each subset of genes including all, secreted genes, genes differentially-expressed in the bleomycin rat model and a combination of secreted and differentially-expressed genes. Four models were constructed in total, and these are available as serialized R objects in our code respository.

## Results

### Feature selection and model construction

We chose the LTRC and NJH cohorts for model training and initial testing, respectively, because they represented distinct patient populations, but were processed on the same expression platform (Affymetrix) by the same authors [9]. These two cohorts also each contained a relatively large number of samples, which is ideal for training and testing statistical models.

**Fig 1:**
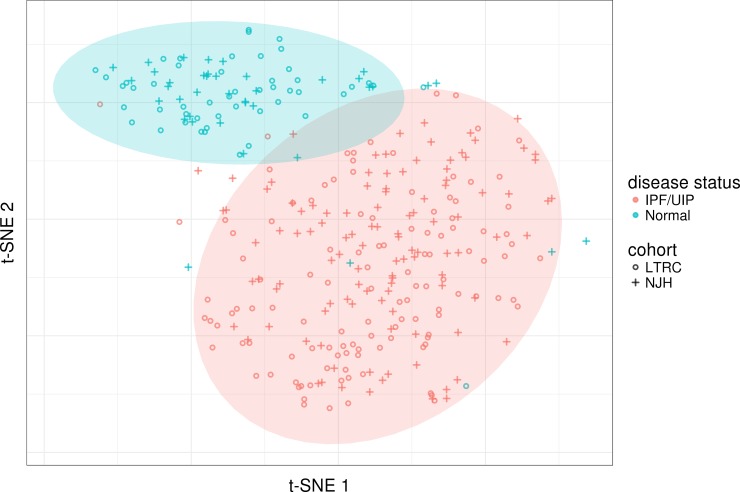
t-SNE models each high-dimensional observation into just two dimensions such that similar observations are modeled by nearby points and dissimilar objects are modeled by distant points. Applying t-SNE to our clinical samples from the LTRC and NJH, We observe distinct grouping of IPF and normal samples with a few outliers. There does not appear to be any grouping of patients by cohort.

Before training, we compared patients to one another in an unbiased manner with t-Distributed Stochastic Neighbor Embedding (t-SNE), a nonlinear dimensionality reduction method capable of reducing the entire transcriptome signal into just two (or three) dimensions for visualization [36]. With transcription data for all IPF and normal patient samples in the LTRC and NJH cohorts, we observed distinct grouping of patient samples by disease status with no clear trend indicating a grouping by cohort (Fig 1, S2 and S5 Figs). A few outliers were identified with this method, but were not excluded from the subsequent work.

The LTRC lung tissue expression data was used to train all models (see Materials and Methods). Four models were constructed in total based on different gene subsets as input. These include models built with all genes (M, 13896 initial features), secreted genes ($M_{secreted}$, 910 initial features), genes differentially-expressed in the bleomycin rat model ($M_{bleomycin}$, 1677 initial features) and the intersection of secreted and differentially-expressed genes ($M_{secreted⋂bleomycin}$, 210 initial features) (S1–S3 Tables). 15 gene features were selected by M (Table 3).

**Table 3:** 15-gene signature for IPF.

Coefficient	Accession	Symbol	Description
-0.3644650	54829	ASPN	Asporin
1.0505567	875	CBS	Cystathionine-beta-synthase
0.3145191	1131	CHRM3	cholinergic receptor muscarinic 3
0.0221471	114805	GALNT13	Polypeptide N-acetylgalactosaminyltransferase 13
0.2791872	374378	GALNT18	polypeptide N-acetylgalactosaminyltransferase 18
0.0460736	2878	GPX3	Glutathione peroxidase 3
0.0214608	4047	LSS	lanosterol synthase (2,3-oxidosqualene-lanosterol cyclase)
0.0028236	56922	MCCC1	methylcrotonoyl-CoA carboxylase 1
0.0747452	8972	MGAM	Maltase-glucoamylase
-0.0382877	4316	MMP7	matrix metallopeptidase 7
0.0546881	5028	P2RY1	purinergic receptor P2Y1
-0.1286685	6423	SFRP2	secreted frizzled related protein 2
0.3537117	25777	SUN2	Sad1 and UNC84 domain containing 2
0.0437323	10579	TACC2	transforming acidic coiled-coil containing protein 2
-0.0245404	64393	ZMAT3	zinc finger matrin-type 3
-10.1203104	(Intercept)	NA	NA

Gene features selected by elastic nets defining the IPF gene signature when no genes have been filtered, using M. The coefficients are extracted from M. Accessions are Entrez gene identifiers.

**Fig 2:**
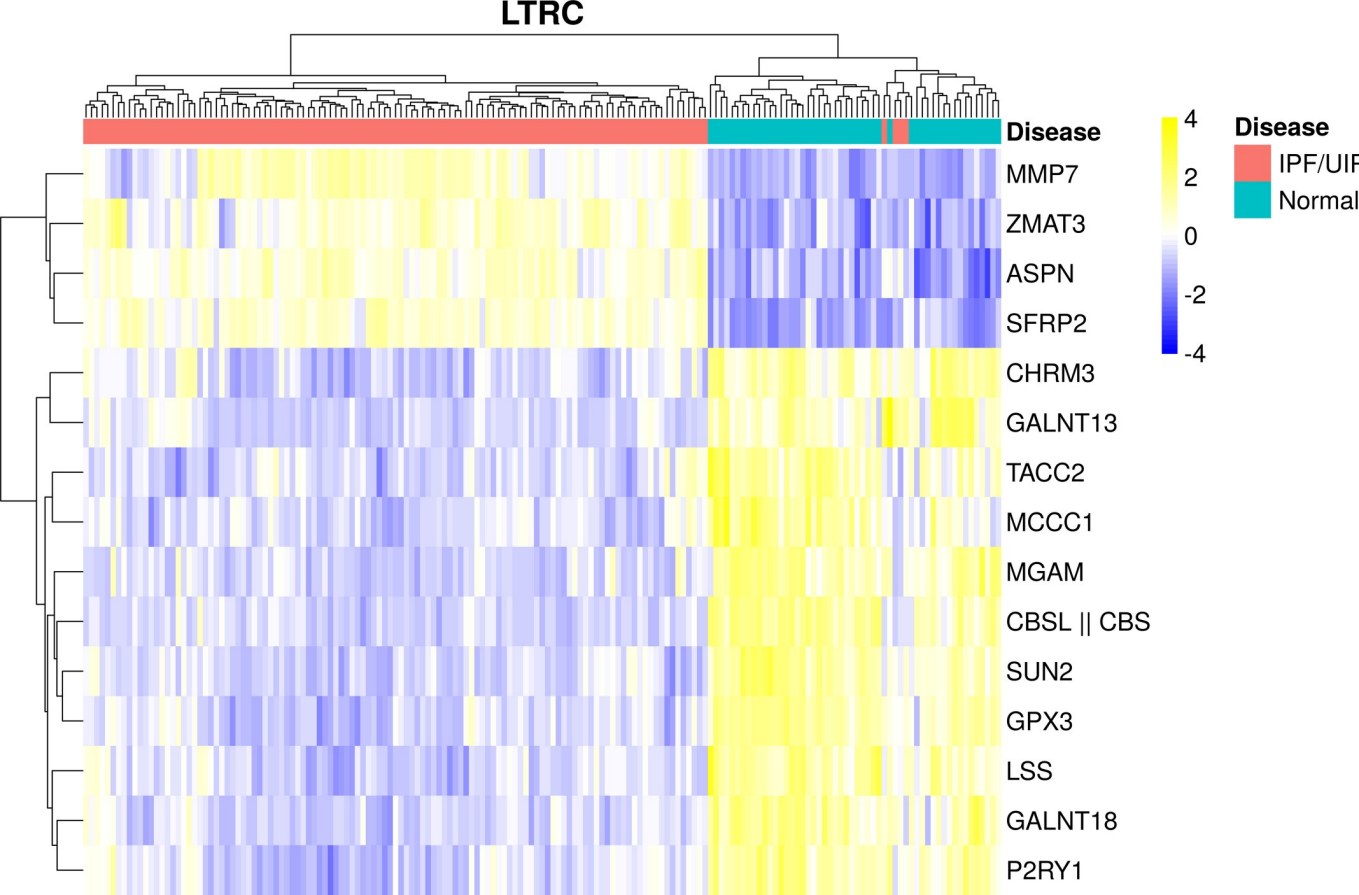
Hierarchical clustering of 15 gene signature used by model M to classify disease status. A clear separation is observed between IPF and normal patient samples. Note that this is just for visualization purposes, and M uses logistic regression to classify samples, not clustering. Row-scaled log intensity units are plotted. We use the complete linkage method for hierarchical clustering with a Euclidean distance measure.

When the expression of these 15 genes is hierarchically-clustered, we observe a very clear separation between IPF and normal patient samples (Fig 2 and S6–S9 Figs). While clustering is not used for disease status classification, the use of this orthogonal data-driven approach independently demonstrates that the 15 gene panel can be used to effectively discriminate between IPF and normal using transcript abundance alone.

### Model validation on independent clinical cohorts

**Fig 3:**
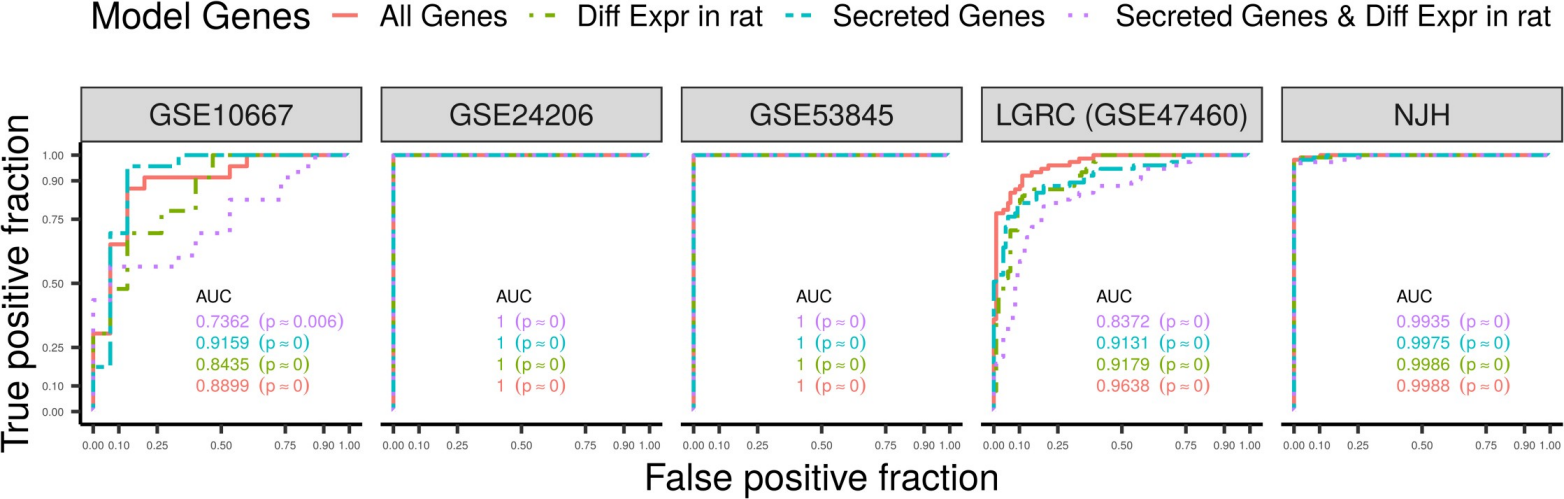
Model performance is assessed on the test data and evaluated with Receiver Operating Characteristic (ROC) curves. We compute the area under the curve (AUC) for each model and each cohort, where a perfect classifier has an *AUC* = 1 and a random classifier has an *AUC* = 0.5 (the diagonal line). The ROC curves and AUCs were calculated by passing the true positive fraction (the probability of a test positive among the diseased population) and false positive fraction (the probability of a test positive among the normal population) to the plotROC package (S5 Table) [38]. We performed 1000 bootstraps of the data for each cohort to establish a null distribution yielding a mean AUC of approximately 0.5, as expected. Based on the empirical p-values from these bootstrap AUCs, we find that all our reported AUCs are statistically significant, confirming the performance of our models across all test cohorts (S4 Table).

We first validated all models ($M,\;M_{secreted},\;M_{bleomycin},\;M_{secreted⋂bleomycin}$) on the NJH cohort. Due to the identical platform and processing, the NJH cohort provided a novel patient sample set while reducing variance from technical factors. All models were also tested on four other independent cohorts (Fig 3). As expected, the most unbiased model (no subsetting of genes before regularization), M, performed the best, while reducing the number of genes for subsequent regularization generally reduced performance. Based on the area under the curve (AUC) metrics, M is the most performant published model of IPF disease status [13,19,37].

### Identifying the most congruent rat bleomycin model

The IPF signatures derived from each of our four models could be used to identify the preclinical rat bleomycin model with the highest congruence to IPF. Before comparing ortholog expression across species, we attempted to normalize species-specific expression by comparing ratios from rat to human, computed as *log*_2_(*bleomycin*/*saline*) for rat samples and *log*_2_(*IPF*/*control*) for human samples. During initial comparisons between rat and IPF using the M feature set, we noticed that many rat genes were not differentially-expressed (|*log*_2_(*bleomycin*/*saline*)|≈0), introducing noise when computing similarity between rat and IPF expression. Therefore, when comparing rat to IPF, we used the $M_{bleomycin}$ feature set (30 gene features), which includes only genes that were differentially-expressed at any of the days in the bleomycin time course. Similarity was computed using Pearson correlation between each day post-bleomycin treatment and IPF samples (using the LGRC samples, to compare our results more directly with those previously published [13]). We found model-IPF congruence increased from days 3 to 14 with maximum similarity between the model and IPF at day 21 (S3 Fig). It is important to note that multiple other murine models of pulmonary fibrosis exist [39], and we have only chosen the rat bleomycin model to compare to the disease, but other model comparisons may be the subject of future work.

## Discussion

Given the challenges associated with the diagnosis of IPF and the inaccuracy of clinical prediction tools, it is imperative to explore new methods for diagnosis, classification and patient stratification. We have effectively leveraged microarray data from a large cohort of IPF patients within the LTRC to generate a new computational classifier of IPF disease. Although IPF disease signatures have been described before [9,13,19,20], the strength of our approach is the number of samples used, the unbiased computational model developed to define the signature and the extensive validation across multiple IPF cohorts. Our model outperforms several other previous models based on the near 100% prediction of disease status across multiple validation cohorts. *Bauer et al*. (2015) described a 12-gene signature identified from about 100 IPF samples compared with control lungs and established the commonality of this signature with that derived from the rat model of bleomycin induced fibrosis at the 7-day time point. Our study complements and extends these findings by developing alternate signatures and establishing congruence with the rat model of bleomycin induced fibrosis. Tissue and peripheral gene/protein expression signatures provide complex information that could be poorly or incompletely understood in the absence of effective computational modeling. Our study identifies a novel 15-gene signature that accurately predicts IPF disease status (Table 3). The signature contains several genes previously not associated with IPF as well as genes such as MMP7 which is a known biomarker for IPF [10,11] and sFRP2, a Wnt-signaling molecule described as a prospective therapeutic target [40]. Notably, MMP7 knockout mice do not develop fibrosis in response to bleomycin treatment [41]. Also, active MMP7 has been detected in IPF lungs but not healthy lungs and has been implicated as a profibrotic metalloprotease [42,43]. Glutathione Peroxidase-3 (GPX3) identified in our signature has been shown to be present in the epithelial lining fluid in the bleomycin-induced fibrosis model and upregulated in IPF [44].

Peripheral blood-derived biomarkers and expression signatures are more clinically translatable and developable as diagnostic tools as opposed to tissue-derived signatures, especially in diseases like IPF where tissues are hard to obtain and gene expression patterns are spatially restricted within the tissue. Profiling of plasma proteome in IPF has identified minimal protein signatures of IPF, as well as potential biomarkers [10,11,45,46] of disease progression including MMP1, MMP7, and surfactant protein-D. In a recent study [47], a 52-gene signature was developed from gene expression profiling of peripheral blood mononuclear cells from a cohort of IPF patients and validated for outcome prediction across two additional cohorts. Many of the identified genes were involved in defense response, wound healing and protein phosphorylation. In our study, we generated a 29-gene secreted protein signature from the tissue microarray data. This signature is enriched for genes in immune response and cell-matrix interaction pathways. Additionally, several extracellular matrix genes such as COMP, SPOCK1, Laminin C1 and ECM2 were identified as signature genes in our study. A secreted protein signature from tissue derived expression data could represent a robust and specific reflection of disease status. Future studies should validate the protein-level expression of these genes in serum/plasma.

In our study, we also show that the rat bleomycin model at day 21 has the highest congruence to the human IPF signature. This contrasts with the results of *Bauer et al*. (2015) wherein the rat model of fibrosis day 7 was determined to be the most similar to human disease. This is likely due to our similarity being assessed only using the IPF-derived gene signatures and not larger sets of genes (S3 and S4 Figs). We determined that using 30 genes to define similarity is more informative than using the entire set of genes that are differentially expressed in IPF and mapped to rat. After day 21, similarity is reduced, but remains relatively high, suggesting a persistent fibrotic state.

In future work, we propose to predict disease progression or severity of IPF with the inclusion of FVC or DLCO lung function measures. This would be analogous to the PROFILE study where *Maher et al*. (2017) showed that a 4 serum biomarker panel could be used to predict mortality and distinguish between stable and progressive IPF [48]. We also note that endpoint gene expression measurements represent a functional vignette of a biological system. Having access to gene expression changes over time along with protein abundances among other measures would shed more light on mechanisms behind IPF, and this is the subject of future work.

We have discriminated effectively between IPF and control lung tissues which is relevant in translational models of disease, but the complexity in diagnosing IPF manifests largely in distinguising it from other idiopathic interstitial pneumonias (IIPs) [49]. While the LGRC contains gene expression from lung samples of control and IPF patients, it also contains COPD and other IIP samples. However, given the paucity of similar data sets, it is challenging to validate a model trained to discriminate between COPD and respiratory bronchiolitis-associated interstitial lung disease or desquamative interstitial pneumonia. Promising modeling efforts are underway, but are limited by the number and diversity of available patient samples (eg. 115 samples across 14 pathology diagnoses, with most diagnoses matching very few patients) [50]. For future work, given the appropriate training and test data, we propose to construct models similar to our own with the ability to distinguish between IIPs.

## Appendix

### A1. Elastic nets: Logistic regression with a binomial distribution

From the glmnet elastic nets R package [28,31], we define the following:

The response variable takes a value in G=1,2. Denote *y*_*i*_ = *I*(*g*_*i*_ = 1).

We model $Pr(G=2|X=x)+\frac{e^{\beta_{0}+\beta^{T}x}}{1+e^{\beta_{0}+\beta^{T}x}}$,

With the log-odds transformation $log\frac{Pr(G=2|X=x)}{Pr(G=1|X=x)}=\beta_{0}+\beta^{T}x$.

The objective function for the penalized logistic regression uses the negative binomial log-likelihood ${min}_{(\beta_{0},\beta )\in R^{p+1}}-\left[\frac{1}{N}\sum_{i=1}^{N}y_{i}\cdot (\beta_{0}+x_{i}^{T}\beta )-log(1+e^{\left(\beta_{0}+x_{i}^{T}\beta \right)})\right]+\lambda [(1-\alpha )||\beta {||}_{2}^{2}/2+\alpha ||\beta {||}_{1}]$, where the elastic net penalty is controlled by the mixing parameter *α* combining both lasso (*l*_1_, *α* = 1) and ridge (*l*_2_, *α* = 0) penalties. The tuning parameter *λ* corresponds to the strength of the penalty.

We note that due to the presence of multicollinearity in high-dimensional expression data, where *p*≫*n*, regularized regression may be used to construct an accurate disease classifier based on transcript abundance, but each model represents one of many possible models [32]. We establish confidence in an individual model (and set of features) by validating/testing it on multiple independent cohorts.

## Supporting information

S1 Fig Elastic net grid search performance. We iterated over a grid of possible paired α and λ parameters for the elastic net module to determine optimal performance while reducing the number of features to create a minimal gene signature. Minimum classification error can be achieved at any value of α given an optimization for λ. The number of features included is annotated for each pair of parameters. The large red block represents a 0 gene feature model (only including an intercept β0).
(TIF)

S2 Fig PCA dimensionality reduction. The proportions of variance accounted for by each of the first two principal components are indicated in parentheses. In this instance, t-SNE was more informative than Principal Components Analysis (PCA) because PCA yields *n*−1 principal components for an observation matrix of *n*×*p* where *p*≥*n* (*n* is the number of observations and *p* is the number of variables), where the variance is non-uniformly distributed across these eigenvectors. Instead the variance is typically spread across more than the first two or three eigenvectors yielding poorer separation between disease and control patients when only taking these eigenvectors into account.
(TIF)

S3 FigCongruence between bleomycin model and IPF.For the 30 gene expresion signature from $M_{bleomycin}$, the similarity between the rat and IPF expression increased from days 3 to 14 post-bleomycin treatment with maximum similarity at day 21. After day 21, similarity is reduced, but remains relatively high, suggesting a possible fibrotic state. *r* = Pearson correlation coefficient where −1≤*r*≤1, with 1 meaning perfectly correlated and -1 perfectly anticorrelated.(TIF)

S4 FigCongruence between bleomycin model and IPF using all differentially-expressed genes.If we examine only those genes that are differentially-expressed in IPF relative to controls ($\frac{IPF}{control}\leq 1.5$ and *FDR*<0.1), and identify the orthologs in the rat, we do not observe increased similarity at any time point post-bleomycin treatment to suggest maximal congruence with IPF. This motivates the use of a smaller gene expression signature to extract only IPF-relevant gene expression.(TIF)

S5 Fig t-SNE dimensionality reduction for all test cohorts. (TIF)

S6 FigHierarchical clustering of 15 gene signature used by model M to classify disease status for all test cohorts.We use the complete linkage method for hierarchical clustering with a Euclidean distance measure.(TIF)

S7 FigHierarchical clustering of gene signature used by model $M_{secreted}$ to classify disease status for all test cohorts.We use the complete linkage method for hierarchical clustering with a Euclidean distance measure.(TIF)

S8 FigHierarchical clustering of gene signature used by model $M_{bleomycin}$ to classify disease status for all test cohorts.We use the complete linkage method for hierarchical clustering with a Euclidean distance measure.(TIF)

S9 FigHierarchical clustering of gene signature used by model $M_{secreted⋂bleomycin}$ to classify disease status for all test cohorts.We use the complete linkage method for hierarchical clustering with a Euclidean distance measure.(TIF)

S1 TableGene features selected by elastic nets defining the IPF gene signature when only secreted genes are included in $M_{secreted}$.The coefficients are extracted from $M_{secreted}$.(CSV)

S2 TableGene features selected by elastic nets defining the IPF gene signature when only differentially-expressed genes from the bleomycin model are included in $M_{bleomycin}$.The coefficients are extracted from $M_{bleomycin}$.(CSV)

S3 TableGene features selected by elastic nets defining the IPF gene signature when secreted and differentially-expressed genes from the bleomycin model are included in $M_{secreted⋂bleomycin}$.The coefficients are extracted from $M_{secreted⋂bleomycin}$.(CSV)

S4 Table Mean AUC from 1000 bootstraps of the test cohort data. (CSV)

S5 Table True positive fraction (the probability of a test positive among the diseased population) and false positive fraction (the probability of a test positive among the normal population) passed to the plotROC package to plot Fig 3. (CSV)

S1 Supporting Information For each model, we report inclusion frequencies of each gene feature using the method of Meinshausen & Bühlmann [33]. (XLSX)
