# Supplementary figures and images for: A robust data-driven genomic signature for idiopathic pulmonary fibrosis with applications for translational model selection
Source: PLoS One. 2019 Apr 18;14(4):e0215565. doi: 10.1371/journal.pone.0215565 (PMC6472794; doi:10.1371/journal.pone.0215565)

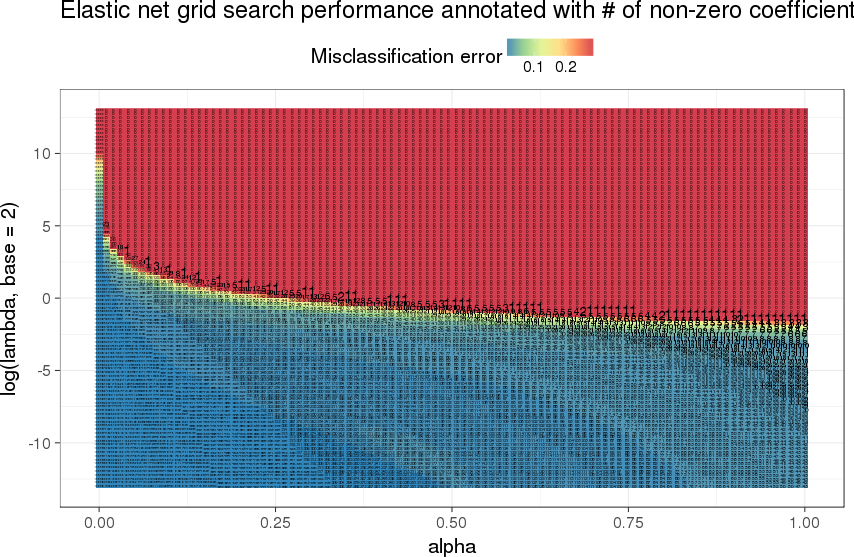

Supplement: S1 Fig — We iterated over a grid of possible paired α and λ parameters for the elastic net module to determine optimal performance while reducing the number of features to create a minimal gene signature. Minimum classification error can be achieved at any value of α given an optimization for λ. The number of features included is annotated for each pair of parameters. The large red block represents a 0 gene feature model (only including an intercept β0). (TIF) [file pone.0215565.s001.tif]

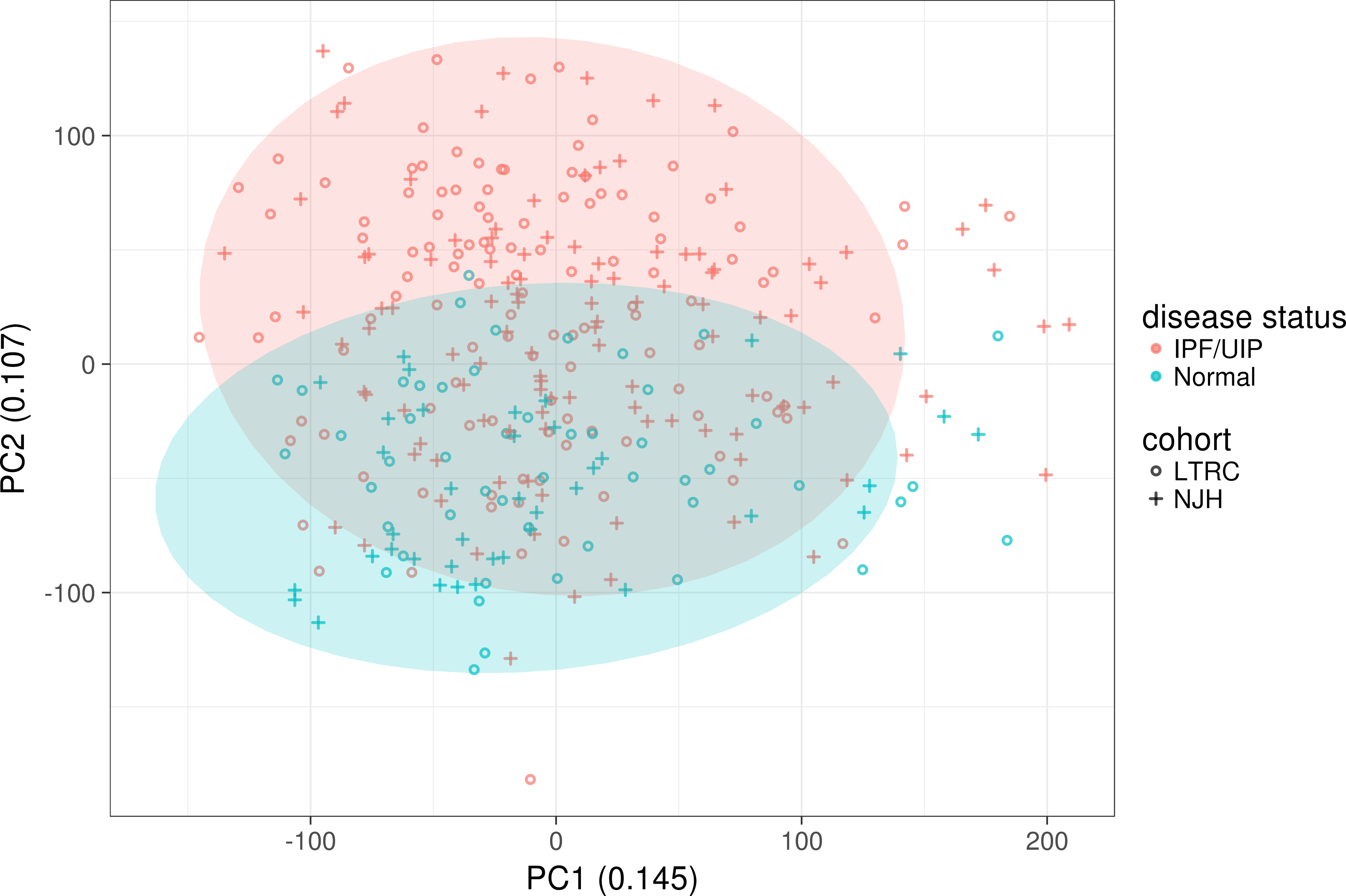

Supplement: S2 Fig — The proportions of variance accounted for by each of the first two principal components are indicated in parentheses. In this instance, t-SNE was more informative than Principal Components Analysis (PCA) because PCA yields n−1 principal components for an observation matrix of n×p where p≥n (n is the number of observations and p is the number of variables), where the variance is non-uniformly distributed across these eigenvectors. Instead the variance is typically spread across more than the first two or three eigenvectors yielding poorer separation between disease and control patients when only taking these eigenvectors into account. (TIF) [file pone.0215565.s002.tif]

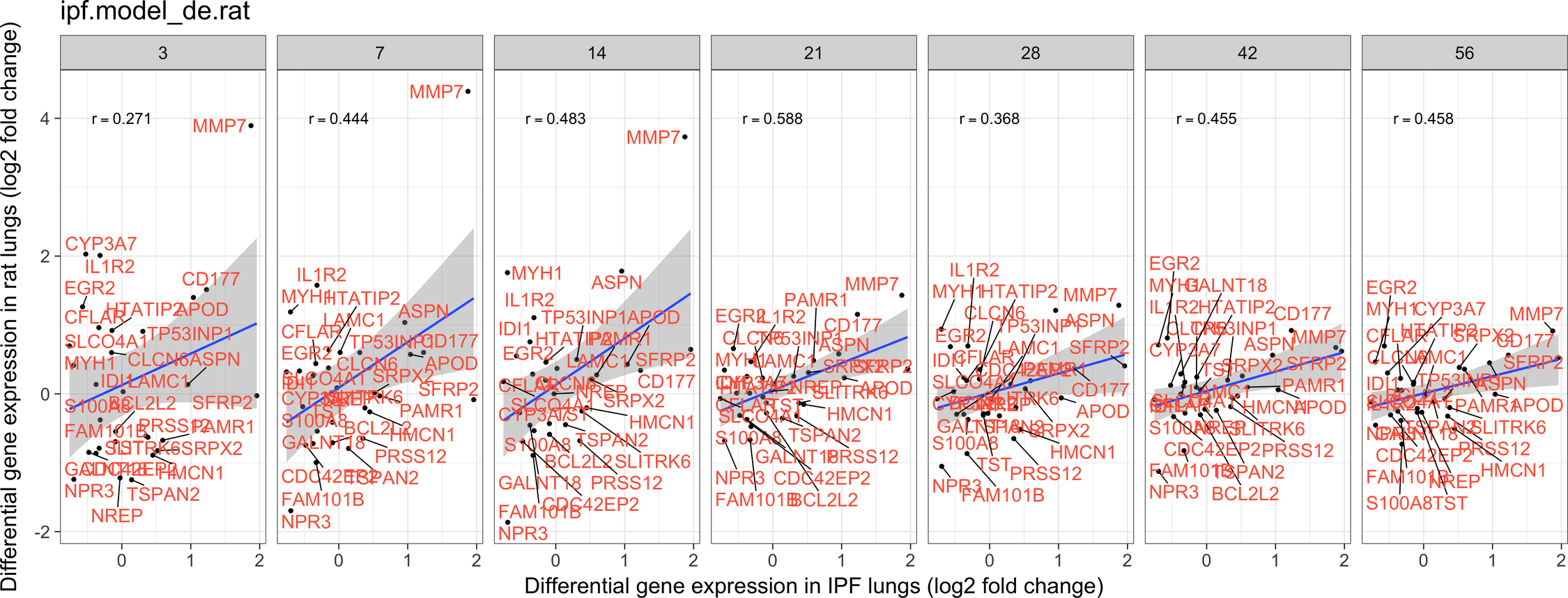

Supplement: S3 Fig — For the 30 gene expresion signature from Mbleomycin, the similarity between the rat and IPF expression increased from days 3 to 14 post-bleomycin treatment with maximum similarity at day 21. After day 21, similarity is reduced, but remains relatively high, suggesting a possible fibrotic state. r = Pearson correlation coefficient where −1≤r≤1, with 1 meaning perfectly correlated and -1 perfectly anticorrelated. (TIF) [file pone.0215565.s003.tif]

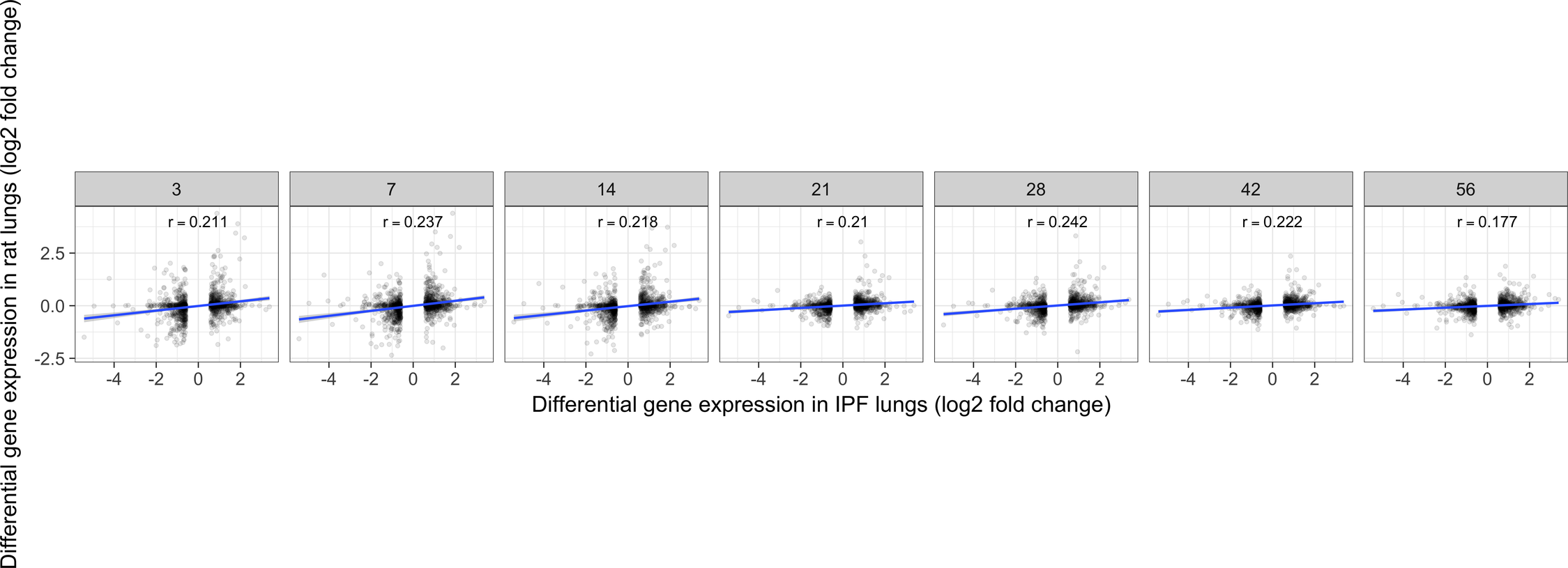

Supplement: S4 Fig — If we examine only those genes that are differentially-expressed in IPF relative to controls (IPFcontrol≤1.5 and FDR<0.1), and identify the orthologs in the rat, we do not observe increased similarity at any time point post-bleomycin treatment to suggest maximal congruence with IPF. This motivates the use of a smaller gene expression signature to extract only IPF-relevant gene expression. (TIF) [file pone.0215565.s004.tif]

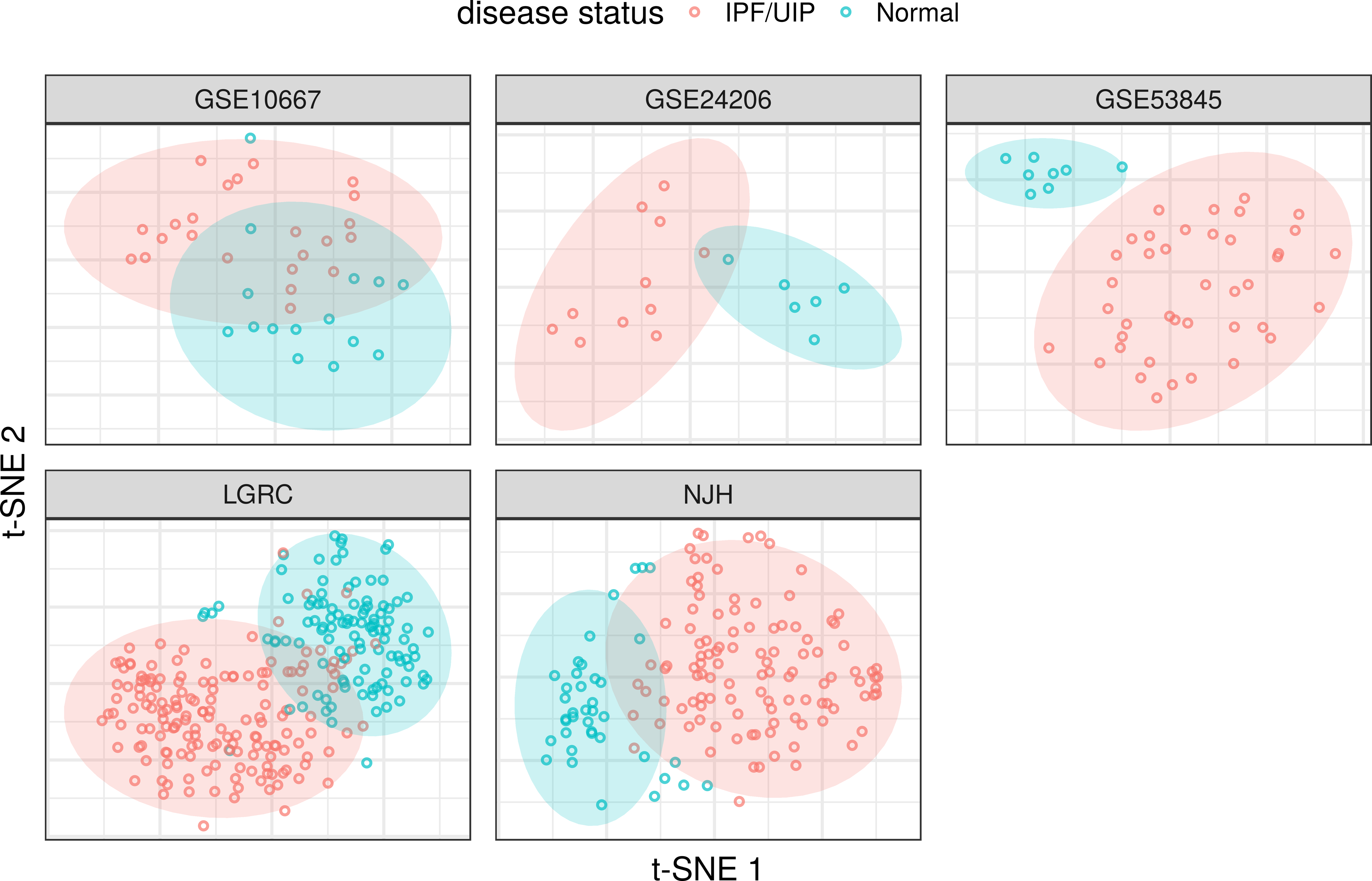

Supplement: S5 Fig — (TIF) [file pone.0215565.s005.tif]

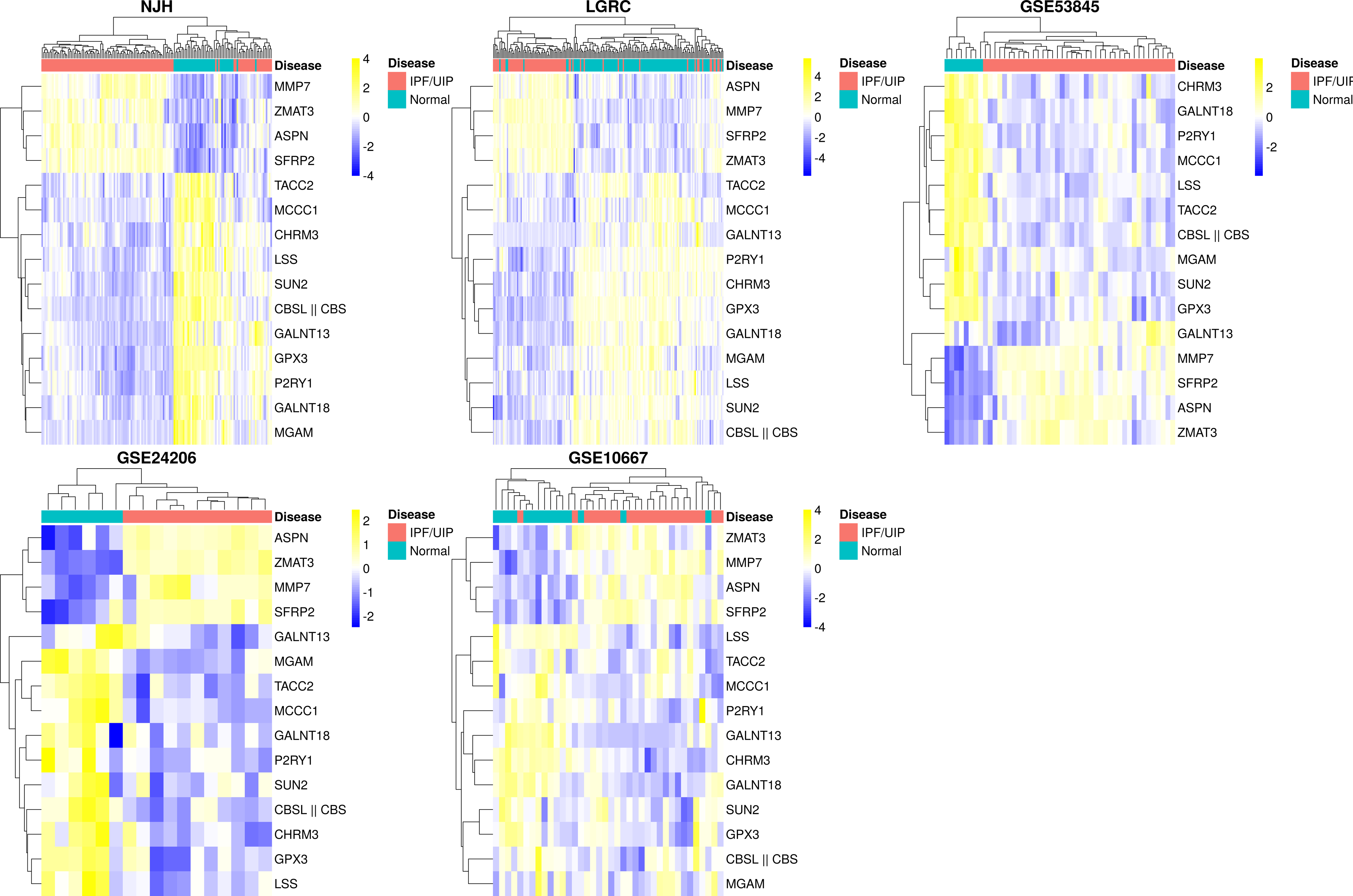

Supplement: S6 Fig — We use the complete linkage method for hierarchical clustering with a Euclidean distance measure. (TIF) [file pone.0215565.s006.tif]

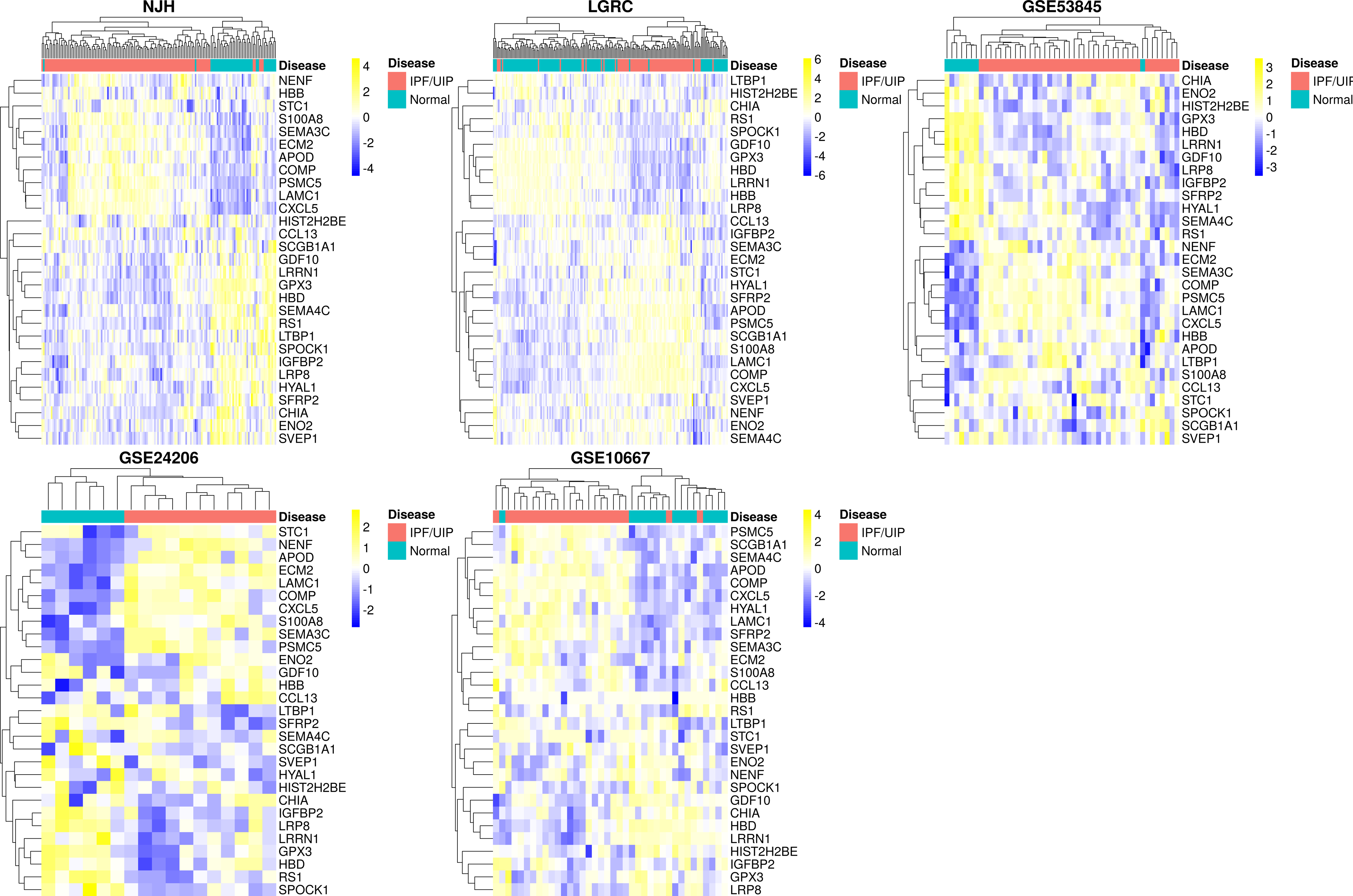

Supplement: S7 Fig — We use the complete linkage method for hierarchical clustering with a Euclidean distance measure. (TIF) [file pone.0215565.s007.tif]

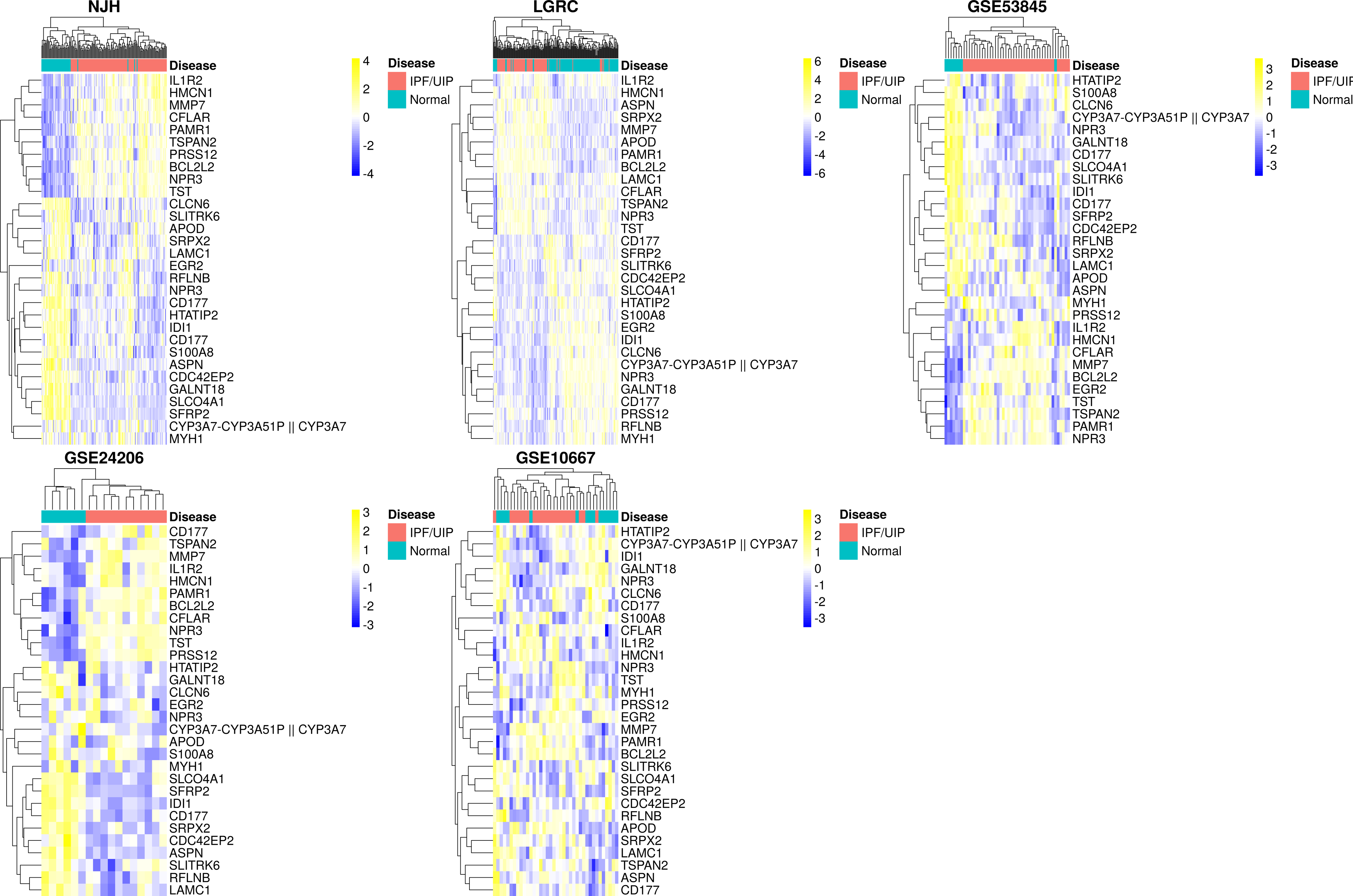

Supplement: S8 Fig — We use the complete linkage method for hierarchical clustering with a Euclidean distance measure. (TIF) [file pone.0215565.s008.tif]

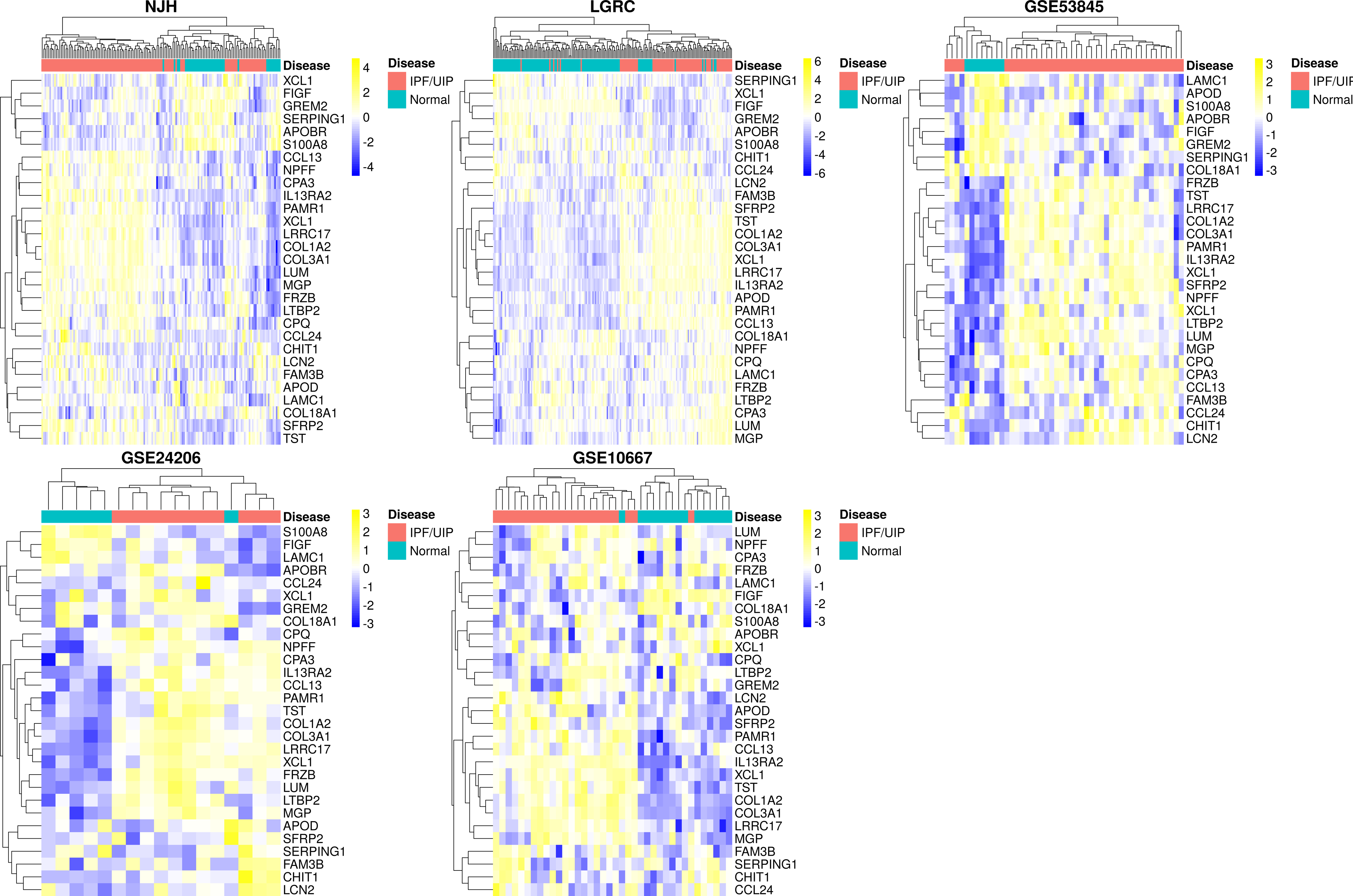

Supplement: S9 Fig — We use the complete linkage method for hierarchical clustering with a Euclidean distance measure. (TIF) [file pone.0215565.s009.tif]
